# Chromatin Compaction Multiscale Modeling: A Complex Synergy Between Theory, Simulation, and Experiment

**DOI:** 10.3389/fmolb.2020.00015

**Published:** 2020-02-25

**Authors:** Artemi Bendandi, Silvia Dante, Syeda Rehana Zia, Alberto Diaspro, Walter Rocchia

**Affiliations:** ^1^Physics Department, University of Genoa, Genoa, Italy; ^2^Nanophysics & NIC@IIT, Istituto Italiano di Tecnologia, Genoa, Italy; ^3^Dr. Panjwani Center for Molecular Medicine and Drug Research, International Center for Chemical and Biological Sciences, University of Karachi, Karachi, Pakistan; ^4^Concept Lab, Istituto Italiano di Tecnologia, Genoa, Italy

**Keywords:** chromatin, nucleosome, coarse-grain modeling, electrostatics, solvation

## Abstract

Understanding the mechanisms that trigger chromatin compaction, its patterns, and the factors they depend on, is a fundamental and still open question in Biology. Chromatin compacts and reinforces DNA and is a stable but dynamic structure, to make DNA accessible to proteins. In recent years, computational advances have provided larger amounts of data and have made large-scale simulations more viable. Experimental techniques for the extraction and reconstitution of chromatin fibers have improved, reinvigorating theoretical and experimental interest in the topic and stimulating debate on points previously considered as certainties regarding chromatin. A great assortment of approaches has emerged, from all-atom single-nucleosome or oligonucleosome simulations to various degrees of coarse graining, to polymer models, to fractal-like structures and purely topological models. Different fiber-start patterns have been studied in theory and experiment, as well as different linker DNA lengths. DNA is a highly charged macromolecule, making ionic and electrostatic interactions extremely important for chromatin topology and dynamics. Indeed, the repercussions of varying ionic concentration have been extensively examined at the computational level, using all-atom, coarse-grained, and continuum techniques. The presence of high-curvature AT-rich segments in DNA can cause conformational variations, attesting to the fact that the role of DNA is both structural and electrostatic. There have been some tentative attempts to describe the force fields governing chromatin conformational changes and the energy landscapes of these transitions, but the intricacy of the system has hampered reaching a consensus. The study of chromatin conformations is an intrinsically multiscale topic, influenced by a wide range of biological and physical interactions, spanning from the atomic to the chromosome level. Therefore, powerful modeling techniques and carefully planned experiments are required for an overview of the most relevant phenomena and interactions. The topic provides fertile ground for interdisciplinary studies featuring a synergy between theoretical and experimental scientists from different fields and the cross-validation of respective results, with a multi-scale perspective. Here, we summarize some of the most representative approaches, and focus on the importance of electrostatics and solvation, often overlooked aspects of chromatin modeling.

## 1. Introduction

If one were to stretch the DNA found inside a cell nucleus, they would end up with an ~2-m long fiber. In order to fit inside the cellular nucleus, which measures ~6 μm in diameter, DNA needs to compact itself in a manner that permits efficient accessibility to DNA-binding proteins, while at the same time reinforcing and compacting the fiber. Compaction is achieved through the wrapping of DNA around certain proteins, the histones, forming the building blocks of the chromatin fiber, the nucleosomes. Nucleosomes are composed of a protein core, the histone octamer (consisting of H2A, H2B, H3, and H4 histone dimers), and 147 base pairs (bp) of DNA wrapped around the core in 1.64 turns. Each histone of the octameric core has a highly disordered N-terminal portion, the histone tail, whereas the rest of the residues form alpha helices (Kalashnikova et al., 2013). Two more tails extend from the C-terminals of H2A histones, amounting to a total of ten unstructured dynamic domains (McGinty and Tan, 2014). Nucleosomes are connected to each other by varying lengths of linker DNA strands, but it has been calculated that the spooling of DNA around nucleosomes alone makes DNA shorter by seven times (Iashina et al., 2017). Chromatin is a molecule that demands multiscale analysis since changes as small as the absence of one DNA bp in nucleosomal or linker DNA can cause non-local changes in the topology of the fiber. Given the fundamental importance of chromatin organization regarding gene expression, the question of discovering the manner in which the genome folds and compacts itself is one of the most fundamental in Biology.

The simultaneous advances in computational and experimental resources not only led to significant milestones, but have also opened new possibilities in chromatin studies. Because of the intrinsic multiscale nature of chromatin, there is a plethora of computational and experimental approaches, which focus on structures as small as the single nucleosome and its dynamics, up to the entire genome of an organism. These models try to describe and predict experimental observables, such as different fiber-start patterns, as well as the effect of different linker DNA lengths on fiber topology. For chromatin modeling, especially at small and intermediate scales, approaches that rely on basic physical interactions for the description of electrostatics and solvation are of uttermost importance. The other indisputably essential ingredient is the mechanical connection; for example, the presence of high-curvature AT-rich segments (A-tracts) in linker DNA is known to influence nucleosome interaction and alter chromatin folding (Buckwalter et al., 2017).

Overall, the study of chromatin is an intrinsically multiscale endeavor, since the effects of interactions spanning from atomic to chromosome-level are both physically and biologically important. Chromatin polymorphism is mostly driven by the delicate equilibrium of electrostatic interactions, solvation effects and mechanical constraints, such as steric exclusion and linker DNA length.

In this review, we provide a succinct synopsis of some among the existing modeling approaches for chromatin, focusing on the physics-based ones, and on those that allow integration with experimental biophysical and/or biological knowledge. This description will be paralleled with that of experimental techniques providing instrumental information for the validation and improvement of these models, paying particular attention on methods that only minimally perturb the observed system. We initially present a variety of chromatin models, starting from works studying single nucleosomes and oligonucleosome fibers, moving on to discuss coarse-grained models and finally fractal models. In the third section of this paper, we examine the fundamental importance of electrostatic interactions in chromatin, and their impact on fiber compaction and polymorphism. This brings us to an exploration of the, often underrated, role of solvation in chromatin compaction, in the fourth section of this review. Finally, we conclude our analysis with a discussion on experimental techniques that have been used in chromatin studies.

## 2. Multiscale Models

**Figure 1:**
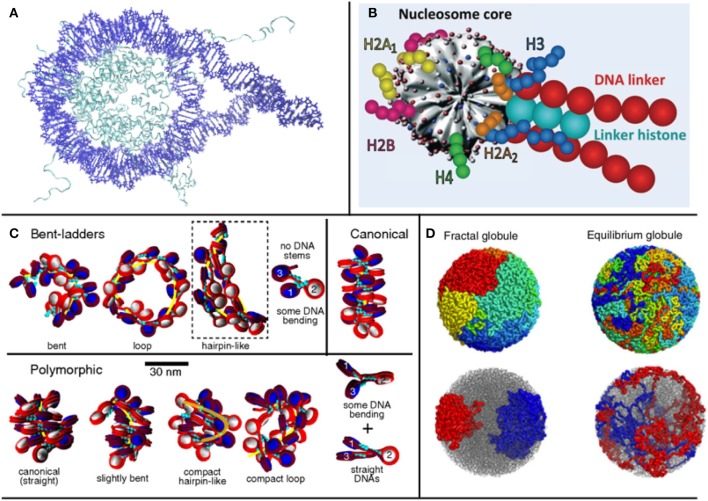
Treating different orders of magnitude in chromatin requires different levels of detail in the representation of nucleosomes: **(A)** for 1-4 nucleosomes, crystal structures can be used (structure used by Shaytan et al., 2016, visualized with VMD); **(B)** for longer structures, a coarse graining model is required [such as Schlick's group model from Collepardo-Guevara and Schlick (2014), used with Permission], **(C)** which can be used to study the topology of oligonucleosome fibers. **(D)** In larger scales, where even the entire genome can be studied, fractal models are used (Mirny, 2011).

Chromatin models can be divided into two general categories, depending on the underlying initial assumptions and on the chosen building blocks: bottom-up and top-down models (Dans et al., 2016). The preferred approach depends on the level of detail of interest, the level of theory that one wants to adopt for the model and, inescapably, the computational capabilities at hand. Bottom-up models take the nucleosome and linker DNA crystal structures as a starting point (Figure 1A). The electrostatics and dynamics of these structures may be studied at the full atom level, and the derived results can be used to feed a coarse-grained model, which allows to draw conclusions for larger systems, such as oligonucleosomes or, sometimes, even larger structures (Figure 1B) (Savelyev et al., 2011; Fan et al., 2013; Collepardo-Guevara and Schlick, 2014; Izadi et al., 2016; Ghosh and Jost, 2018). The parameters used in these coarse-grained models depend on the properties of interest and on those observed by the accompanying experiments. In order to parameterize these types of models, data is often used from all-atom structures and simulations, making their results dependent on the resolution of the structures and the performance of the force fields used.

In top-down models, the behavior of the fiber is deduced from experimental observations and sequencing of large regions of chromatin, or even of the entire genome, from which a scheme of interactions is derived. Given the limitations in resolution and accuracy of experiments, top-down models cannot possess the same level of detail as bottom-up models. However, they provide a way to study global chromatin properties. These models may incorporate a multitude of, often *ad-hoc*, coarse grained descriptions to look into very specific chromatin features related to smaller scale structures, such as the kbp scale. Finally, in this category of models the use of notions from polymer physics is very common, representing chromatin as a polymer chain and its stages of compaction as phase transitions, imposing constraints in the forms of potentials. (Giorgetti et al., 2014; Bianco et al., 2017)

Alternatively (Imakaev et al., 2015), chromatin models have been divided in categories based on whether they are built to match pre-existing data or emerge as representations of physical properties: data-driven models and *ab initio* models. Regarding data-driven models, some examples are given by approaches that try to generate chromosome structures based on Hi-C maps (Fudenberg et al., 2016), translating contact probability to distance. In these cases, however, one needs to bear in mind that Hi-C maps, and sequencing techniques in general, often give an average picture of the genome. *Ab initio* models, on the other hand, take properties that have been observed or even hypothesized about chromatin as a starting point, and aim to reproduce them through the application of constraints and potentials (Tompitak, 2017; Lequieu et al., 2019). The mathematical nature of these models can sometimes lead to a simplification of biological factors at play.

Here, bearing in mind these general classifications, which are consistent with model classifications in many fields, we propose an exploration of various models based on the final order of magnitude that they are able to study, ranging from mononucleosome studies up to works examining the entire genome. Examining different orders of magnitude of chromatin, we present approaches that make use of different assumptions and are based on different types of data, illustrating the multifaceted nature of the topic. An overview of different modeling paradigms based on the order of magnitude at interest is shown in Figure 1.

### 2.1. From the Single Nucleosome to Oligonucleosome Fibers

Nucleosomes have the ability to dissociate entirely in histones and DNA, upon unwrapping, and then reassemble (Kulaeva et al., 2012). The curvature of the DNA can either favor or disfavor histone-DNA contacts, and therefore the formation of nucleosomes (Szerlong and Hansen, 2011). Based on this premise, starting our analysis from the building blocks of chromatin, we encounter Partially Assembled Nucleosome States (PANS), which are interesting as they reveal the electrostatic and mechanical changes that occur when a nucleosome is forming or dissolving. Rychkov et al. (2017) analyzed three types of PANS (hexasomes, tetrasomes, and disomes) through Molecular Dynamics (MD) simulations, visualizing the structures with Atomic Force Microscopy (AFM) experiments. The nucleosome formation procedure was observed to occur as such: the two H3 and H4 dimers bind to the DNA first, forming a tetrasome, followed by the sequential addition of H2A and H2B dimers. The results were compared to Small Angle X-ray Scattering (SAXS), Forster Resonance Energy Transfer (FRET), and AFM data. Nucleosome disassembly follows the reverse order, and both assembly and disassembly were seen to be associated with DNA supercoiling, as a way to regulate torsional stresses on the fiber (Bancaud et al., 2007).

Linker DNA length is extremely important for chromatin compaction, not only for mechanical but also for electrostatic reasons. Determining how linker DNA influences chromatin topology, and how its length and sequence can affect compaction has been the subject of many studies and speculations. In the work of Buckwalter et al. (2017), for instance, the presence of so-called A-tracts, DNA segments where multiple A-T pairs are present in a row, and their influence on DNA rigidity, and therefore on chromatin fiber flexibility, are examined. It has been observed by comparison of MC simulations and Electron Microscopy (EM) experiments on reconstituted oligonucleosome arrays that the presence of A-tracts causes DNA bending angles of up to 90°, and that these particular segments are often found in linker DNA (Cui and Zhurkin, 2009). The direction of bending of the linker DNA is also relevant for compaction: for example, when DNA bends inwards at the exit sites from the NCP the resulting structures are more compact compared to the opposite case, and give rise to zig-zag configurations and closer overall nucleosome proximity. It is evident that linker DNA length is of great importance when it comes to chromatin topology; however, its role is not immediate; the really important parameters for packing are the DNA bending angles, which are influenced by linker DNA length through topological and persistence length constraints.

The presence of the linker histone H1 (or H5 in avian chromatin) is also a key for compaction. This histone is not always present in nucleosomes, and its position can vary on or off the nucleosome dyad axis, the axis of symmetry of the nucloeosome (Pachov et al., 2011). The H1/H5 changes the orientation and flexibility of linker DNA, forming contacts with both entering and exiting strands. When two or more nucleosomes in sequence are bound to H1 histones, rigid structures termed DNA stems are formed, which present straighter linker DNA and reduced separation angle between the entering and exiting DNA; the latter effect is more pronounced in chromatin configurations with long linkers (Collepardo-Guevara and Schlick, 2014). The increased rigidity of DNA because of the formation of DNA stems is mitigated by the dynamic nature of H1/H5 binding and unbinding on nucleosomes (Collepardo-Guevara and Schlick, 2012).

Most all-atom and coarse grained models dealing with chromatin simulations require the use of empirical force fields at some point, impacting on the simulation results. Even though an extensive critical comparison of force fields and force field modifications for nucleic acids is beyond the scope of this review, we suggest the works by Galindo-Murillo et al. (2016) and Dans et al. (2017).

### 2.2. Coarse-Grained Oligonucleosome Models

According to the number of nucleosomes in the start of a fiber, different behaviors have been observed. Among some general categories, the most prominent of which are the zigzag and solenoid fiber models (Buckwalter et al., 2017). Zigzag models for chromatin propose what is commonly called a two-start fiber model (two nucleosomes at the start of the fiber), in which linker DNA crosses the main fiber axis. In two-start zigzag models, nucleosomes are stacked in the periphery of the fiber and linker DNA occupies the central space of the structure. Solenoid models on the other hand propose compaction through coiling of the linker DNA along the superhelical path. In these models, fibers are one-start, and nucleosomes create frontal contacts, with 6 to 8 nucleosomes per turn of the fiber. It is thought that both models coexist in fibers, along with straight linker DNA and bent linker DNA. (Grigoryev et al., 2009; Schlick and Perišić, 2009) Contrary to the zigzag fiber, where the dominant interactions are *n* ± 2, in solenoid models they were found to be *n* ± 5 or *n* ± 6 (Robinson et al., 2006; Grigoryev et al., 2009) where n represents the position of the reference nucleosome.

Besides the number of nucleosomes at the start of the fiber, and taking into consideration the fact that linker DNA length is not always the same across the fiber, different nucleosome repeat lengths (NRL) produce different fiber configurations, and alter the propensity of a fiber to unfold. In Collepardo-Guevara and Schlick (2014), MC simulations were performed on coarse-grained oligonucleosome fibers (Figure 1C) to study these variations, and observed a variety of structures, reaching the—perhaps not surprising—conclusion that structures with highly varying NRL were more compact than uniform structures, a direct consequence of fewer topological constraints. In relation to gene expression, the study also found that transcriptionally active cells presented shorter NRLs, while in inactive cells the opposite was observed (Gilbert et al., 2004). In the coarse-grained model, shorter NRL fibers arranged in ladder-like forms, while medium fibers arranged in zig-zags and longer NRLs resulted in heteromorphic structures (Grigoryev et al., 2009).

Nucleosomes bearing histone modifications, or even less histones than the canonic octamer (Winogradoff et al., 2015) have also been studied as a factor influencing chromatin compaction. In this study by Diesinger and Heermann (2009), a genome folding model was constructed using Monte Carlo (MC) simulations and introducing histone and nucleosome depletion. In a subsequent paper, the role of epigenetic modifications regarding nucleosome depletion was investigated, and MC data was compared to 5C and fluorescence *in situ* hybridization (FISH) (Diesinger et al., 2010). Even though full atom models are very instructive in the mononucleosome scale, in certain mesoscale chromatin models (Kulaeva et al., 2012), DNA base pairs are represented as rigid bodies, with parameters that account for orientation and displacement. Oftentimes, in more coarse-grained models, nucleosomes are treated as rigid bodies with concentrated charge and the dynamics of the histone tails are modeled as Gaussian distributions or as series of beads. Works like Giorgetti et al. (2014); Kepper et al. (2008) model chromatin as an inextensible chain of beads, whose distance depends on the spatial scale of the desired simulations.

Works like Koslover et al. (2010); Koslover and Spakowitz (2009) aim to optimize chromatin morphology through studying its dependence on linker DNA elasticity and length, introducing the role of inter-nucleosome core particle (NCP) interaction potentials in the packing of the fiber. Such works often use MC or Brownian dynamics simulations (Wedemann and Langowski, 2002; Langowski, 2006) and model electrostatic interactions based on potentials at various levels of sophistication. In Koslover et al. (2010), the chromatin fiber is constructed as a helical array by cyclically repeating a fundamental structure, defined as two nucleosomes and the linker DNA between them, in which nucleosomes are treated as rigid bodies and linker DNA as a series of beads. As we mentioned previously, histone modifications are also relevant factors for chromatin compaction, and are sometimes used as model parameters. An example of histone modifications as model parameters is MacPherson et al. (2018), a polymer MC coarse-grained model using methylation as a parameter to study chromatin dynamics and conformation statistics.

In the work of Schiessel et al. (2001), so-called two-angle models were developed, using linker DNA entry and exit angles and NCP twist angles, generating ensembles of minimum energy conformations through MC and analysing their dynamics through Brownian dynamics. NCP geometry becomes itself a parameter in several works (Kepper et al., 2008; Stehr et al., 2008; Kulaeva et al., 2012), in which internucleosomal interactions are specifically studied as triggers for compaction. When it comes to the representation of the NCP as a rigid body, shapes, such as an oblate ellipsoid or an oblate spherocylinder are more accurate than simple spheres. In Kepper et al. (2008), a coarse-grained computer model was applied to a sample pool of 101 nucleosome arrays, using different chromatin models with and without the presence of linker DNA. It was shown that nucleosome spacing is relevant to chromatin stability, with the highest destabilization occurring at a 2 bp shift, by analysing energy landscapes. Energy variations were compared to values from chromatin stretching experiments (Cui and Bustamante, 2000). After surpassing the 2 bp energy barrier, nucleosome repositioning toward a new conformation, rather than returning to the original one, becomes more energetically favorable. Nucleosome orientation was also shown to be of importance, since, for example, it was observed that in cases where a nucleosome was oriented transversally it occupied more volume and caused its neighbors to be pushed further apart, hindering close packing.

### 2.3. Topological and Fractal Models

During the past decade, great progress has been made in the study of chromatin organization due to the advent of Chromosome Capture technologies (3C). The field was particularly revolutionized by Hi-C, which provides the interaction frequencies between loci of an entire genome. 3D reconstructions of genomic regions and even entire genomes are possible, using Hi-C data, through structural inference and statistical methods (Lesne et al., 2014; Varoquaux et al., 2014). There are two main categories of techniques to generate 3D structures from Hi-C contacts: ensemble approaches and consensus approaches. In the latter case, the Hi-C data are considered as a single ensemble, while in the former models different categories of structures are created from the data. It has been suggested recently that it might be possible to reconstruct the diploid 3D chromatin structures (Cauer et al., 2019).

It can be of interest to combine results from high throughput techniques, such as Hi-C, with computer simulations. In Ohno et al. (2019) parallels were drawn between protein structure and chromatin. Through a combination of Hi-C data at nucleosomal resolution obtained at several cell phases and coarse grained simulations, Ohno et al. observe two general secondary structure types in chromatin, which they call α-tetrahedron and β-rhombus, as an analogy to the α-helix and β-sheet structures in proteins, supporting the claim that fibers can alternate between these structures when nucleosome positioning changes. Information on nucleosome orientation was gleaned through analysis of the spatial proximity between DNA entry and exit points in individual nucleosomes across the genome and their 3D positioning. Solvation effects were not directly taken into account, as nucleosomes were modeled as space-filling objects, and linker DNA was also implicitly treated.

In the study of compaction and larger scale interactions within the chromatin fiber, for example for characterizing the Topologically Associating Domains (TADs), loop extrusion models are very significant. TADs are regions of the genome with enhanced contact frequency, identifiable on Hi-C maps as squares. During loop extrusion, Loop Extrusion Factors (LEFs), such as cohesin, interact with chromatin, inducing the formation of loops until they encounter a Border Element (BE), such as CTCF. It has been observed by Goloborodko et al. (2016) that macroscopic loop characteristics depend on the abundance of LEFs. Loop extrusion models provide explanations for experimental observations, such as the preferential orientation of CTCF, the enrichment of TAD boundaries in proteins with architectural functions, and TAD merging in LEF deletion experiments, and could provide insight on chromosome-level phenomena (Fudenberg et al., 2016). Polymer simulations are frequently used by loop extrusion models to make predictions and to validate analytical models. Loop formation has also been studied with mesoscopic models, where it was observed to depend on linker histone presence, ion concentration, and linker DNA length (Bascom et al., 2016). In addition to the “one-sided” loop extrusion mechanism described above, recent research indicates that “two-sided” loop extrusion might prove to be more robust in explaining experimental data (Banigan et al., 2019).

In the last decade, there has been growing interest on fractal models describing chromatin, and part of the chromatin modeling community, particularly emerging from polymer physics, has been focusing on the possibility that chromatin organizes itself as a fractal, especially since a similar state has been proposed in the seminal paper of the Hi-C method by Lieberman-Aiden et al. (2009). In this work, a distinct case of the previously theorized globular equilibrium model was proposed for the Mbp scale: the fractal globule—otherwise called crumpled globule (Grosberg et al., 1988), a polymer conformation that enables maximally dense packing while preserving the ability to easily fold and unfold any genomic locus (Lieberman-Aiden et al., 2009; Mirny, 2011; Tamm et al., 2015) (Figure 1D). In such models, as in polymer models for chromatin in general, chromatin is considered as a flexible polymer fiber, and the notion of the single nucleosome is lost. Because of their large scope, these kinds of models can be relevant for large scale systems or even the entire genome.

Distinct chromosomal regions can be modeled as equilibrium globules, structures used to describe polymers in poor solvent conditions (Lieberman-Aiden et al., 2009). The chromatin fiber could assume a Peano curve conformation, which represents a continuous fractal trajectory that densely fills space without crossing itself. In fractal globules, compaction is achieved through the collapse of the globule and it has been shown that the fractal globule has the ability to organize territorially, alluding to chromosome territories, (Tamm et al., 2015) distinct regions in the nucleus occupied by certain chromosomes, in contrast with the previously proposed equilibrium globule, which does not present such organization. In the fractal globule, the number of interactions as a function of volume shows a linear correlation, which leads to the interdigitation of different regions in the globule with each other, allowing for extensive genomic cross talk (Mirny, 2011) (Figure 1D). This is particularly interesting for two main reasons: cross talk has been observed in simulations between the regions, and fractal globules unfold in an optimal way, which is relevant in the study of transcription.

However, it needs to be noted that the fractal globule is a metastable state, unlike the equilibrium globule, and that its lifetime depends on topological constraints, which, in real cells, can be affected by enzymes and DNA-binding proteins. Fractal globules have been observed experimentally in Hi-C experiments (Lieberman-Aiden et al., 2009; Rao et al., 2014; Ghosh and Jost, 2018) and Small Angle Neutron Scattering (SANS) experiments (Ilatovskiy et al., 2012; Iashina et al., 2017). The relationship between the physical environment of a fractal chromatin fiber and transcription has been studied in several works, such as Almassalha et al. (2017), in which the analytical correspondence between changes in the fractal dimension of the chromatin fiber and increment of chromatin accessibility and compaction heterogeneity was studied. Furthermore, the authors speculated that differences in the transcription of a certain gene might be influenced by folding of neighboring genomic regions. The findings were supported by microscopy measurements on cancer cells.

Fractal globule models have been criticized based on the argument that self-similarity cannot be assessed in only a couple of orders of magnitude. However, researches in the field, such as Bancaud et al. (2012) claim that, even though mathematical fractals are self-similar ad infinitum, physical fractals are only self-similar within certain orders of magnitude, typically 2 or 3, while chromatin architecture spans 4 or more orders of magnitude, and a common fractal architecture would connect all of them under a single topological theme, without the need for separate structures in each order of magnitude.

To conclude this section, we present a summary table (Table 1) of the models mentioned, categorized by the final order of magnitude that they treat (e.g., single nucleosomes, oligonucleosome arrays, entire genome). We include information on the computational methods used, and when available, the type of experimental data used for result validation.

**Table 1:** Computational and experimental works mentioned in this review (partial account), listed under the scale of interest.

**Scale**	**Publication**	**Subject**	**Computational techniques**	**Experimental data**
NCP	Fan et al. (2013)	Ionic dependence of aggregation	Langevin MD	FCS
	Materese et al. (2009)	Ion condensation, NCP solvation	MD, PBE	PDB structure
	Buckwalter et al. (2017)	Sequence dependence of DNA curvature	MC	EM
	Rohs et al. (2009)	Sequence dependence of DNA electrostatics	PBE	PDB structures
	Shaytan et al. (2016)	Histone tail interaction	MD	
	Rychkov et al. (2017)	NCP Assembly	MD	SANS, FRET, AFM
	Davey et al. (2002)	NCP Solvation		NMR
	Luger et al. (1997)	Nucleosome structure		X-ray crystallography
	Bertin et al. (2004)	Histone tail interaction		SAXS
Nucleosome arrays	Diesinger and Heermann (2009); Diesinger et al. (2010)	Histone and nucleosome depletion	MC	FISH
	Collepardo-Guevara and Schlick (2014)	NRL-produced patterns	MC	
	Kepper et al. (2008)	NRL-produced patterns	MC	Stretching experiments
	Norouzi and Zhurkin (2018)	Nucleosome array unwrapping	MC	FCS
	Stehr et al. (2008)	Inter-NCP interactions	MC	Data from various techniques
	Gan and Schlick (2010)	Ionic dependence of aggregation	PBE, DiSCO	Data on DNA bending
	Grigoryev et al. (2009)	Linker histones, ionic dependence	MC	EM
	Izadi et al. (2016)	Electrostatics, histone tails, linker DNA	PBE	Cryo-EM
	Koslover et al. (2010)	Linker DNA	Energy optimization	EM, FCS
	Bascom et al. (2016)	Loop formation	MC	3C
Entire genome	Ohno et al. (2019)	3D genome architecture	Hi-CO method	Hi-C
	Cauer et al. (2019)	Hi-C 3D reconstruction	Mathematical modeling	Hi-C
	Lieberman-Aiden et al. (2009)	Fractal globule	Polymer simulations	Hi-C
	Iashina et al. (2017)	Fractal globule	Mathematical analysis	SANS
	Ghosh and Jost (2018)	Chromosome modeling	Coarse-grained Polymer model	Hi-C
	Fudenberg et al. (2016)	TADs	Polymer model	Hi-C
	Giorgetti et al. (2014)	TADs	Polymer model	3C, FISH
	Bianco et al. (2017)	TADs	Polymer model	5C
	Ricci et al. (2015)	Nucleosome aggregation		STORM
	Le Gratiet et al. (2018)	Chromatin organization in the nucleus		CIDS, Fluorescence

*Computational techniques and experimental data used are listed, when applicable*.

## 3. Electrostatic Interaction in Chromatin: An Intrinsically Multiscale Phenomenon

At large scales in the chromatin fiber, structures are approximately electrostatically neutral, allowing for an average treatment of electrostatics and solvation in polymer models for chromatin. However, at the NCP and oligonucleosome scale, electrostatics and solvation become extremely important, due to the high charge of the DNA. The charges present on the DNA backbone are partly neutralized by the winding of DNA around the histone core, especially through the effect of the histone tails, and partly through counter-ions present in the nuclear environment. The modeling of internucleosomal interactions using reductionist analytical potentials, which omit the explicit role of histone tails, can cause secondary, but still relevant, electrostatic effects to be overlooked.

Considering the biological importance of different ionic types, Mg^2+^ is particularly significant, as it has been found to promote nucleosome condensation and aggregation and could promote linker DNA bending, because in its presence interactions of first and third neighboring nucleosomes are boosted (Grigoryev et al., 2009). Tetravalent cations on the other hand require lower concentrations to induce compaction (Zinchenko et al., 2017). In Fan et al. (2013), systems of 1–10 nucleosome core particles (NCPs) were studied using a coarse-grained model in order to study the effects of monovalent, divalent, and trivalent cations on these structures, reproducing experimental data. It was observed that an increase in K^+^ ions amplified the repulsive internucleosomal electrostatic interaction; increasing Mg^2+^ concentration caused partial aggregation, and an increase in COHex^3+^ ions triggered a strong mutual internucleosomal attraction in 10 NCP systems, therefore showing that the aggregation of NCPs is different under the effect of different types and concentrations of counterions.

Multivalent ions and the effect of their distribution around NCPs on chromatin conformation were also studied in Gan and Schlick (2010), using a mean-field Poisson Boltzmann Equation (PBE) approach, with an emphasis on shielding charges, which aggregate particularly around DNA and the exposed parts of the histone tails. The fact that a surface needs to be exposed to solvent in order for ions to bind on it makes ion-caused electrostatic screening (a change in the effective electric charge) and ion-chromatin interactions in general directly dependent on compaction. Calculations showed that the enhanced screening due to divalent ions might not only be because of their higher charge, but also because they form a denser layer of counterions around the NCP and fluctuations in this layer are correlated to different fiber conformations. This makes even more evident the fact that the topology of compaction is a key determinant for chromatin-ion interaction. It was observed in these simulations that the shielding charge arising from both monovalent and divalent ions was linearly correlated with the ionic strength of the solution.

In the study of structures as large and complex as chromatin, it has been proposed in Izadi et al. (2016) that implicit solvent Generalized Born (GB) simulations would be preferable to traditional fully explicit MD, in order to circumvent computational limitations. However, standard GB scales poorly with the number of solute atoms and, in this work, a multiscale atomistic GB model that incorporates improvements in the electrostatic calculations is presented, the accuracy of which was evaluated through point-by-point comparison with PBE calculations. Taking advantage of the natural hierarchical organization and charge distribution of chromatin, Izadi et al. used approximate point charges to calculate electrostatic interactions between distant points in a 40-nucleosome structure, containing ~1 million atoms, focusing particularly on the behavior of the histone tails. They were able to reproduce experimental findings of the interaction of the H3 histone tail and the linker DNA. The GB approach proved the existence of viable alternatives that drastically reduce the cost of conformational sampling in very large structures.

One could not conclude a discourse on chromatin electrostatics without mentioning the effect of the histone tails, which have been found to promote stability of the linker histone on the NCP. In some models, histone tails are modeled as a series of beads with one positive charge per bead (Gan and Schlick, 2010; Fan et al., 2013; Korolev et al., 2014). It was seen by Shaytan et al. (2016) that certain histone tail configurations promote DNA bulging at entry and exit sites, possibly contributing to the formation of twist defects in the nucleosomal DNA. Twist defects are DNA deformations that allow for one more or less DNA bp in positions where DNA interacts closely with histones (Brandani et al., 2018). They are important, among other reasons, because their presence causes the formation of nucleosomes with 146 bp instead of the usual 147 (Pasi and Lavery, 2016), due to overwinding and stretching of the DNA (Davey et al., 2002). They also speculated that the presence of arginines and lysines might impose constraints on histone tail motion because of attractive electrostatic interactions. Contacts between DNA and histones were seen to be dominated by the histone tails, making up 60% of protein-DNA interactions in the nucleosome, rapidly wrapping around the DNA (in Shaytan et al., 2016, it was observed that they do so in the first 20 ns of the simulation).

In another study, the N-terminal of the H4 histone tail was observed to interact with the “acidic patch” present on the surface of adjacent nucleosomes, a small groove formed by eight residues, six belonging to H2A and the remaining to H2B, which constitutes a region of highly negative charge density on the nucleosome surface, serving as a hot-spot for DNA-binding proteins and histone tails (Kalashnikova et al., 2013; McGinty and Tan, 2014; Zhou et al., 2018). Throughout 1 μs-long MD simulations in Shaytan et al. (2016), the NCP is seen to be very stable in dynamics, in contrast to histone tails and linker DNA: large scale unwrapping or opening of NCP DNA were not observed, even when simulations were performed in 1M salt concentration, under which conditions they are known to occur (Wilhelm et al., 1978). This indicates that such phenomena might take place on longer time scales. Of particular interest are the histone H3 tails, which have been suggested by experiments (Kato et al., 2009) to form stable folded structures, and even to potentially compete with other DNA-binding proteins, affecting accessibility of epigenetically modified sites in the minor grooves.

It has already been mentioned that the presence of A-tracts can change the curvature of DNA, causing the minor grooves to be narrower than those in segments with lower curvature, and locally enhancing negative electrostatic potentials. In Rohs et al. (2009), PBE calculations were performed on DNA, showing that the electrostatic potential caused by the DNA backbone had intensity peaks inside the major and minor grooves. The position of these peaks correlates with the positions of arginine residues on the histone core. Previously observed binding preference for arginines over lysines in minor grooves, and especially in narrower ones, was partly explained via a combination of electrostatic and desolvation effects. For the study of minor groove geometry, all the crystal structures of protein-DNA complexes containing at least one base atom–aminoacid contact were analyzed. Analysis of nucleosomal DNA was based on the nucleosome structures available on the Protein Data Bank (PDB) at the time.

## 4. The Role of Solvation in Chromatin Compaction

The role of the solvent in biomolecular interactions is known to be crucial. In part, this is because of solvent-mediated electrostatic effects—the screening of the water molecules and that of the ions in solution. In addition, there is the so-called cavity formation phenomenon, which penalizes the occurrence of solvent-excluded regions. Chromatin spatial arrangement, due to NCP charge, size and porosity, is expected to be particularly affected by these phenomena, which must be accurately considered. It has already been described that the formation of the fundamental unit of chromatin, the nucleosome, is carried out by the complexation of the negatively-charged DNA polymer with the positively-charged histone protein octamer. If investigated at the molecular level, this process is governed by a number of interactions, such as hydrogen-bonds, salt-bridges, and water-mediated interactions occurring along the positively-charged arginine anchors that intercalate deep inside the minor grooves of DNA facing the histone core (McGinty and Tan, 2014; Gebala et al., 2019). When it comes to histone core-DNA electrostatic interactions, it is known that every nucleosome presents 14 non-covalent histone-DNA contacts, at the sites of arginine residues (Szerlong and Hansen, 2011).

Solvent exposure affects electrostatic interactions at the nucleosome level: compared to H3 and H4 histones, the two H2 variants are more solvent exposed, making them more accessible to chromatin-binding proteins as well (Izadi et al., 2016). Specific ion binding sites and their location on the nucleosome are also of particular interest, and they can be studied using electron density maps in combination with chemical information (Davey et al., 2002). It has been observed that sodium preferentially condenses around regions rich in solvent accessible acidic residues, especially in areas with two or more acidic residues in close proximity (Materese et al., 2009). It is also speculated that, in chromatin fibers exhibiting high compaction, internucleosomal electrostatic repulsion could be reduced in intensity because of an increased neutralization of the DNA backbone charge by the neighboring histone cores and counterion screening.

The idea that the nucleosome is an impermeable object has been proven erroneous (Materese et al., 2009); in this work, it was seen that mobile ions are able to reach the NCP inner core because of high levels of local solvation (more than 1,000 water molecules). This led to the conclusion that the local value of dielectric constant in the region facing the histone core is larger than expected. The authors also looked into the mobility of water molecules on the first hydration layer of the nucleosome and, as expected, found them to be less mobile than bulk water molecules. Through detailed visualization of structured water at the protein-DNA interface, they also found that water molecules not only contribute significantly to the stability of DNA binding but also adapt histone surfaces to conformational variations of DNA, facilitating nucleosome dynamics. All-atom electrostatics calculations were conducted and compared to PBE calculations, observing a slight inconsistency between the two. PBE predicts that the most significant contribution to DNA charge neutralization comes from the enhancement of the electric field and that it is a result of the tight wrapping of the DNA around the histone core. These results indicate that close condensation of ions around the nucleosome can significantly reduce the short range effect of the nucleosomal charge, having as a natural consequence the facilitation of chromatin close packing.

In another work concerning NCP solvation (Davey et al., 2002), the solvent-accessible surface area (SASA) of nucleosome crystals with 147 and 146 bp was investigated. NCPs with 147 bp were found to possess a SASA of ~74 Å^2^, which is distributed mostly in the cavities within the histone octamer and in the space between it and the DNA. The primary hydration layer of the NCP was found to contain slightly more than 2,000 water molecules, the positions of which were found to largely correspond to the positions of A-tracts, especially in the vicinity of the minor groove. Water was shown to be important in the two main mechanisms of protein-DNA recognition: direct readout (nucleotide chemically specific bonds) and indirect readout (sequence-dependent conformational features of DNA recognized by sterically complementary protein contacts). Structures termed “spines of hydration” were also observed, in which water molecules bind regularly to adenine N3 and thymine O2 atoms (Kopka et al., 1983). Structural analyses have shown that the phosphate groups are the most strongly solvated components of the DNA (Egli et al., 1998; Schneider et al., 1998).

**Figure 2:**
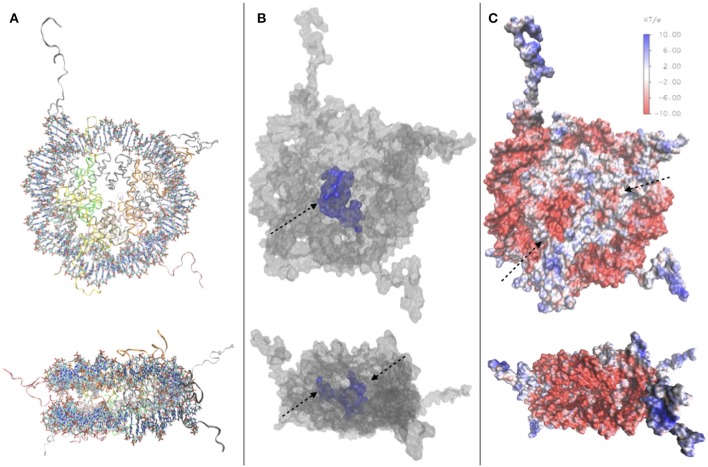
**(A)** Top and side view of the 1kx5 crystal structure. **(B)** Top and side view of the SES of 1kx5, constructed with NanoShaper (Decherchi and Rocchia, 2013) and visualized via VMD. The channel traversing the histone core is represented in blue together with an adjacent open cavity and is indicated by an arrow. On the side view, the entrance and exit of the channel can be seen, indicated by arrows. **(C)** Electrostatic map of the SES of 1kx5. Areas of negative surface potential are indicated in red and areas of positive surface potential in blue. The acidic patch is indicated by an arrow on the histone core. Another minor acidic region, composed by fewer residues on the surface of histone H4, is also highlighted. Most of the exposed regions of the histone core are electrically neutral, with the acidic patch representing the main exception. Remaining positive charges of the histone core are buried, due to the binding of encircling DNA. We also note positive charges on the histone tails, and strong negative charges on the DNA backbone.

In order to illustrate the porosity of the nucleosome, particularly described in Materese et al. (2009), we have conducted a study on the nucleosome crystal structure [PDB code 1kx5 (Davey et al., 2002), Figure 2A] using NanoShaper interfaced with VMD (Decherchi and Rocchia, 2013; Decherchi et al., 2018), providing the values of the Surface to Volume Ratio (SVR), the number of cavities and pockets. We measure an SVR of 0.387 Å^−1^, which reflects a quite high porosity (Shirota et al., 2008), and a number of cavities and pockets. In Figure 2C, we visualize the channel traversing the nucleosome core, which significantly impacts on NCP accessibility to water and ions. Our results are consistent with previous qualitative analyses mentioned in this section, and indeed indicate that the nucleosome is highly solvated and porous. We have also constructed an electrostatic map of the nucleosome, using data from the DelPhi Poisson-Boltzmann solver Rocchia et al. (2001) on the potential and constructing the SASA of the nucleosome with NanoShaper (videos of the full 3D structure found in Supplementary Material), as seen in Figure 2B, where it is possible to clearly see, among other features, the position of the acidic patch (residues E56, E61, E64, D90, E91, E92 of H2A and E102, E110 on histone H2B (Kalashnikova et al., 2013), and the highly charged histone tails, both key elements in chromatin compaction and chromatin interaction with DNA-binding proteins. This analysis showed a minor acidic region, on the surface of histone H4.

## 5. Experimental Studies of Chromatin: From the Nucleosome to the Nucleus

Throughout this review, we have highlighted the main manifestations of the multiscale nature of chromatin, and we have explored the multitude of factors affecting its compaction. The interplay between simulations and experiments is crucial to reach a deep understanding of this complex system, and has given rise to breakthroughs that would have been impossible without the combination of the two approaches. Experimental investigations of chromatin can be carried out at different scales, similarly to computational approaches. Having already mentioned some experimental results validating computational models, we have specifically looked into some of the experimental techniques used in both small and large scales, from the NCP up to entire nucleus.

**Figure 3:**
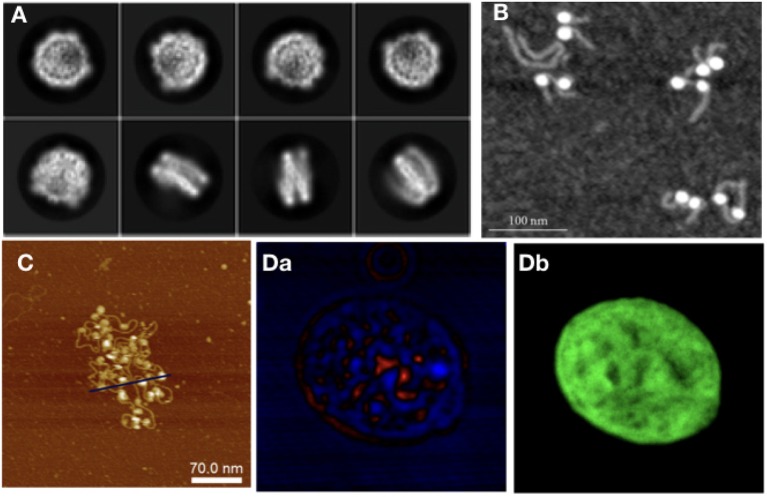
As with modeling approaches, in experiments different techniques are required to study different orders of magnitude in chromatin: **(A)** NCP imaged with Cryo-Em (adapted from Kobayashi et al., 2019), **(B)** NCPs with histone tails AFM image (Filenko et al., 2012), **(C)** Nucleosome array, AFM image (adapted from Krzemien et al., 2017), **(D)** Isolated Hek nucleus imaged with CIDS **(a)**, labeled with Hoechst for chromatin-DNA organization imaging. The fluorescence labeling **(b)** is used as a fingerprint of chromatin to demonstrate the correlation with the label-free approach using circular polarization excitation (Le Gratiet et al., 2018).

Starting from the nucleosome, experiments have been carried out to determine its crystal structure, with continuing endeavors starting from Luger et al. (1997), in which a 2.8 Å resolution structure of the NCP was obtained via X-ray crystallography, using reconstituted nucleosomes. In Luger's work, many of the structural elements of the nucleosome were uncovered, such as the number of base pairs wrapped around the octamer, which were unknown despite the fact that the octamer histone structure had already been observed. The histone tails and their structural role have also been studied to great extent in Widom (1997). Since then, further structures with 147 bp (Davey et al., 2002) and 146 bp (Tachiwana et al., 2010) have been observed. The study of sub-structures, such as the histone tails and of site-specific interactions (van Emmerik and van Ingen, 2019) in more detail, required the use of NMR (Davey et al., 2002). In latest years, there has been growing interest for the study of NCPs using Cryo-EM (Figure 3A). The sample preparation protocols involved in this technique make it an interesting alternative to X-ray crystallography for structural studies. Cryo-EM provided information on custom-made NCPs in studies relevant to DNA binding protein-NCP interactions (Takizawa et al., 2018) and also on interactions of the NCP with components of the nuclear environment, such as the nuclear pore complex (Kobayashi et al., 2019). The orientation of NCPs has also been observed by Cryo-EM in a recent study, where it is stated that in the most common arrangement of a pair of NCPs they are placed in parallel, facing histone octamers (Bilokapic et al., 2018).

X-ray crystallography provides structures with atomic resolution, which are key for atomic-level studies. However, this approach has some limitations; it fails to provide good information on the more mobile domains of the NCP, and it cannot be used for large oligonucleosomes (the largest structures that have been crystallized to date are tetranucleosomes (Schalch et al., 2005; Ekundayo et al., 2017). In order to circumvent these constraints, one can turn to scattering techniques. SAXS studies have looked into the issue of whether the histone tails protrude into the solvent surrounding the NCP or associate with DNA at physiological salt conditions. The histone tails are notoriously hard to resolve in crystallography because of their size and intrinsically mobile nature (Kato et al., 2009; Zhou et al., 2012; Gao et al., 2013). Using SAXS however, it is possible to indirectly observe whether the histone tails are solvated or adherent to the DNA, by measuring changes in the overall structure size. Bertin et al. (2004) have applied SAXS to study histone tails as well, focusing on the structural details of internucleosomal interactions and the effects that histone tails have on them. Often SAXS has been used in conjunction to other techniques to correlate structural to dynamical data. In Mauney et al. (2018) SAXS, FRET, and MD were used to dissect the sequence-dependent DNA unwrapping mechanism. Fluorescence Correlation Spectroscopy (FCS) has been used in a work by Fan et al. (2013) to estimate NCP stacking energy. In this combined experimental and theoretical work, model parameters were tuned based on comparison with single molecule FCS and SAXS data, which also showed that histone tails facilitate NCP stacking by acting as bridges between NCP surfaces. FCS data was also used by Norouzi and Zhurkin (2018) to tune the parameters of an MC model of nucleosome arrays under the influence of external forces.

Moving on from NCPs to larger structures, nucleosome arrays are the next step; besides SAXS (Howell, 2016), AFM has also been used to study arrays of varying lengths (Figure 3B). The advantage of using this technique for chromatin is 2-fold: there is the possibility of taking many measurements, making it good for statistical purposes; and it allows for the study of electrostatic and related interactions, such as differences in ionic concentration. The importance of ionic interactions with chromatin has naturally gained the attention of the experimental community. Studies, such as Gan and Schlick (2010) have shown that Mn^2+^ ions bind to the major DNA groove near CG pairs. In Krzemien et al. (2017) AFM was used to measure the changes in chromatin topological conformations depending on salt levels in the environment (Figure 3C). Studying NCP arrays in varying salt concentration revealed that array compaction has a non-monotonic salt dependence. Increasing salt concentration induces partial screening of the charges of the DNA backbone, therefore reducing the electrostatic interactions between DNA and histones, directly impacting on compaction. The stability of mononucleosomes has also been investigated in correlation with salt concentration (Hazan et al., 2015): in low to intermediate salt regimes they observed some partially disassembled states (as also studied computationally in Rychkov et al. (2017) where H2A/H2B histone dimers partially dissociate from the NCP. Regarding the mechanical properties of chromatin, DNA stiffness was observed to be salt-dependent as well, in accordance with other experimental and computational studies (Rohs et al., 2009; Pasi et al., 2015, 2017; Pasi and Lavery, 2016); the persistence length was seen to increase at higher ionic concentrations.

Optical microscopy, a field traditionally tied to biological applications, is a natural candidate for chromatin studies, due to the advances in resolution obtained by super-resolution techniques, and to the fact that label-free optical microscopy methods have been on the rise for the past decade. Experiments using the single molecule super-resolution microscopy technique STORM (Ricci et al., 2015) have observed units of chromatin organization termed by the authors *clutches*, heterogeneous groups of various sizes. The size of the clutches has been speculated by Ricci et al. to be related to the pluripotency capacity of each cell, and the median number and nucleosome density in the nucleus was found to be cell-specific. From longer nucleosome arrays to chromatin fiber, other super-resolution techniques, such as Photoactivated Localization Microscopy (PALM) have been used to extrapolate chromatin topology in the nucleus from nucleosome dynamics. Label-free techniques are also used to study chromatin at the nuclear level, such as Circular Intensity Differential Scanning (CIDS) in Le Gratiet et al. (2018). In this work, it is shown that the main advantage of this polarimetric method compared to standard fluorescence microscopy is the capability to obtain specific contrast mechanisms due to the chiral organization of the DNA in a label-free approach without a priori knowledge of the sample. Indeed, it is shown that the stronger signal region corresponds to more compacted DNA region, i.e., heterochromatin, while the weaker signal, such as for the nucleoli, corresponds to a lower compaction, i.e., euchromatin region (Figure 3D).

Experimental validation has been attempted also for some among the most exotic theoretical models proposed for chromatin, namely those hypothesizing fractal globules. Fractal globules have been observed experimentally in Hi-C experiments (Lieberman-Aiden et al., 2009) and Small-Angle Neutron Scattering (SANS) experiments (Ilatovskiy et al., 2012; Iashina et al., 2017). The important question tackled by works on this topic is the way in which fractal states with stable long-lived properties are formed. SANS has been considered a good technique for experiments looking for fractal structures in the nucleus because of its extended spatial range, from ~15 nm to 10 μm. The use of Cryo-Electron Tomography (Cryo-ET) has provided insight on the structure of mitotic chromosomes in fission yeast (Cai et al., 2018). SAXS and Cryo-EM have also been used in structural analysis of the fiber up to the chromosome level (Figure 3A) (Joti et al., 2012; Nishino et al., 2012; Maeshima et al., 2014, 2016).

## 6. Conclusions

Chromatin is an extremely complex system, the behavior of which is tuned both by mechanical and electrostatic factors, and by biological interactions. Simulations provide extremely useful insights, depending on the level of approximation used to represent the system, on the different mechanisms and factors influencing compaction. In this review we mention several computational works that used as inputs parameter sets acquired through experiments or evaluated their results by comparing them with preexisting experimental data. It is clear that combining simulations results with various experimental techniques, appropriate to the resolution of interest, can help shed light on the main determinants of chromatin compaction.

Electrostatics in chromatin encompasses an intricate combination of different mechanisms and the importance of its role in compaction and chromatin remodeling is paramount. The high negative charge of DNA is partially neutralized by the direct interaction of the latter with histones (including the effects of histone tails and the linker histone), but electrostatic stabilization of the chromatin fiber is achieved through a combination of this effect with long-range electrostatics and solvent screening. Simulations in which ionic interactions with chromatin at the NCP level are treated more accurately would be a great improvement to existing approaches. In addition, a more accurate representation of the nucleosome core is crucial when performing these analyses, since solvation has proved to be a very important factor in nucleosome behavior, whereas neglecting these effects would hamper a correct understanding of chromatin compaction.

In summary, we have presented an overview of some, mostly theoretical and computational, approaches to the description of chromatin, from the nucleosomal to the cellular level, particularly focusing on the role of electrostatics and solvation as the driving mechanisms of chromatin conformational changes and equilibria. To complement this overview, we also presented some representative experimental approaches to study chromatin structure and dynamics, at both small and large scales.

## Author Contributions

AB performed most of the reviewing and writing tasks. SD and AD organized and discussed the experimental part. SZ worked on the analysis of the role of solvation. WR and AB decided the organization of the manuscript and checked the consistency of the work. All authors reviewed and checked the manuscript.

### Conflict of Interest

The authors declare that the research was conducted in the absence of any commercial or financial relationships that could be construed as a potential conflict of interest.

## Supplementary Material

Supplementary Video 1 View of the full 3D surface of the nucleosome core particle (PDB code 1KX5), coloured by electrostatic potential. Blue regions indicate positive surface potential, while red regions indicate negative potential.

Supplementary Video 2 View of the full 3D Solvent Excluded Surface Area of the nucleosome core particle (PDB code 1KX5). Blue regions indicate the channel traversing the histone core, and an adjacent large cavity.
